# Metalloids: Semi as Metals yet Full of Antimicrobial Potential

**DOI:** 10.1002/cmdc.202500979

**Published:** 2026-04-18

**Authors:** Kevin Böhm, Muhammad Jawad Nasim, Claus Jacob

**Affiliations:** ^1^ Division of Bioorganic Chemistry School of Pharmacy Saarland University Saarbrücken Germany

**Keywords:** antibiotic, antifungal, antimicrobial, antiparasitic, metalloids

## Abstract

In the periodic table, the metalloids boron (B), silicon (Si), germanium (Ge), arsenic (As), antimony (Sb), and tellurium (Te) demarcate a borderline between the metals and non‐metals. Also referred to as semi‐metals, their behaviour is indeed characterized by inorganic ions, organometallic complexes, and covalent compounds. Yet despite this unique medley of inorganic and organic molecules, reactivities and activities, and century‐old traditional uses, their pharmaceutical applications today are often underestimated or associated with outright toxicity. Still, recent research has taken a closer look at (some of) these elements and has encountered an amazing assortment of natural metalloid‐containing products, many with distinct antimicrobial activities. At the same time, metalloid‐containing complexes have been synthesized and tested successfully as ionophores, in ligand‐exchange reactions with amino acids, such as cysteine, or as redox modulators triggering oxidative stress. This has been complemented by an extensive synthesis of organic metalloid‐based compounds, even including bioisosteric replacements for carbon. Today, a few metalloid compounds are still or already used in therapy; others are in clinical trials, and many more are in the pipeline, clearly demanding full attention for the semi‐metals and their many colourful compounds.

## Graphical abstract

Metalloids such as boron (B), silicon (Si), germanium (Ge), arsenic (As), antimony (Sb), and tellurium (Te) bridge metals and non‐metals, displaying unique chemical versatility. Recent research highlights their diverse antimicrobial and therapeutic potentials, from natural products to synthetic organic and organometallic molcules, underscoring renewed interest in these once‐overlooked semi‐metals.

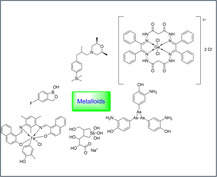

## 1 Introduction

The sharp increase in antimicrobial resistance represents one of the most urgent pharmaceutical challenges of our time and necessitates the search for novel antimicrobial substances [1, 2, 3]. In the course of this search, a closer look should also be taken at elements that are currently rather neglected in drug development, such as the metalloids, a couple of main group *p*‐block elements nested in the periodic table between the metals and the non‐metals.

Indeed, the metalloids, or as they are sometimes called ‘semi‐metals’, represent a very special group of elements in the periodic table and commonly include boron (B), silicon (Si), germanium (Ge), arsenic (As), antimony (Sb), and tellurium (Te). Together, they form a diagonal borderline between the metals to their left and non‐metals to their right. Bismuth (Bi), polonium (Po), and astatine (At) are also sometimes included, as they would be the logical followers in row 6 of the periodic table, yet these elements are de facto very metallic rather than semi‐metallic, and in any case, with the possible exception of bismuth, play a very minor role in chemistry and the life sciences. As semi‐ or ‘half’‐metals, and not to be confused with the *d*‐block transition metals, the classic metalloids indeed exhibit various properties usually associated with metals, including a metallic appearance in some of their elemental modifications and the ability to form alloys, yet also share some of the typical non‐metallic features, such as forming covalent bonds with carbon, nitrogen, oxygen, sulfur, and other elements [4, 5]. Other properties such as conducting heat and electricity, electronic band structure, ionization energy, and electronegativity fall between the metals and non‐metals [4, 6]. Therefore, it is not unusual to find these elements at the centre of some typical inorganic chemistry, for instance as charged ions or in complexes, yet also in quite stable covalent organic – or shall we say *organometallic* – compounds.

Intriguingly, these elements and their compounds have also found their ways into the life sciences not only as poisons but also as traditional medicines. The activities of some of them, such as the ones based on arsenic, are legendary and indeed such metalloid preparations have been used in medicine for thousands of years across the globe and still are today. Others, such as germanium, are more exotic yet still worth considering.

In this review, we shall therefore briefly explore the potential and current uses of metalloids in Medicine, with a focus on the antimicrobial activities associated with these elements and their various compounds. Apart from looking at more traditional applications of elements such as arsenic, boron, and antimony, we shall explore the specific molecular mechanisms and biochemical mode(s) of action associated with such metalloid molecules. Eventually, we shall try to answer the question if there is anything ‘special’ in the chemistry of semi‐metals which may endow their compounds with specific properties, reactivities, and activities possibly not found in the case of ‘real’ metals or non‐metals.

## 2 Boron: Everything but Boring

In the periodic table, the first metalloid encountered is boron, the fifth element, located in the second row and framed between the distinctively metallic beryllium (Be) on the one side and carbon (C) on the other. As expected for such a semi‐metal, boron shows metallic as well as non‐metallic properties. On the one side, it forms ions (in oxidation state +3) and complexes, yet also various covalent compounds, for instance with carbon. Thanks to its electronegativity of 2.04, such organic boron compounds tend to be fairly stable. Since boron only has three valence electrons, reaching a full shell octet in covalent compounds tends to be tricky, and very unusual chemical structures such as three‐centre two‐electron systems, are not uncommon. From a purely chemical perspective, such exotic molecules may indeed drive you ‘bananas’, yet fortunately they do not play any notable role in Biology.

In Nature, boron occurs in its ionic B^3+^ form, usually as boric acid B(OH)_3_ or esters of borate [B(OH)_4_]^−^. Boric acid has historically been used as an antiseptic, with its antiseptic properties largely due to its acidity [7]. Because of its toxicity, predominantly in infants, its application as an antiseptic has been discontinued [8]. Unlike typical oxoanions, such as phosphate (PO_4_^3−^), borate [B(OH)_4_]^−^ behaves more like a metal complex and does, for instance, readily exchange HO^‐^ ligands for other organic or inorganic ligand anions, as we shall see in the case of boromycin and other natural boron‐containing molecules.

**FIGURE 1:**
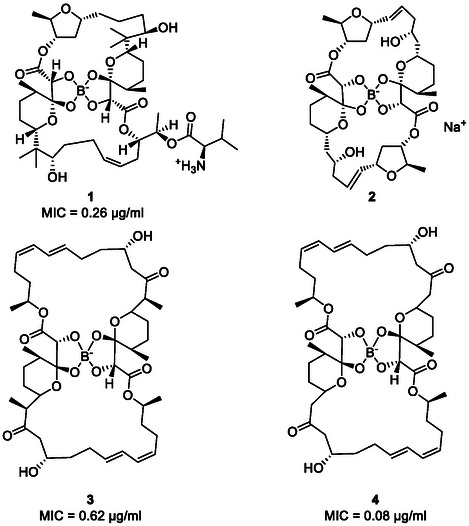
Structures of boromycin (**1**), aplasmomycin (**2**), tartrolon B (**3**), and tartrolon E (**4**) and corresponding MIC values against methicillin susceptible *S. aureus*. For compound **2,** no MIC is reported. Nafcillin, as a first‐line treatment for severe infections caused by methicillin‐sensitive *S. aureus*, provided a MIC value of 0.5 µg/ml [10, 11, 12, 13].

Thanks to the complex chemistry of borate, natural boron compounds are not that uncommon either, and the first boron‐containing natural product found by Huetter and coworkers in 1967, namely boromycin, even happens to be an antibiotic [9]. Boromycin (**1**; Figure 1) is a polyether‐macrolide produced by *Streptomyces antibioticus* and shows antibiotic effects against Gram‐positive bacteria, activity against HIV, and protozoan parasites [14]. Mechanistically, boromycin acts as an ionophore which causes the loss of potassium ions, resulting in cell cycle arrest and eventually cell death. The boric acid moiety appears to be essential for binding the ions, while a loss of the D‐valine moiety does not affect the activity [10, 15]. Since the discovery of boromycin, other boron‐containing ionophores have been isolated from several soil bacteria, such as aplasmomycin (**2**) from *S. griseus* SS‐20 and tartrolon B (**3**) from the cellulose‐degrading myxobacterium, *Sorangium cellulosum* [16, 17]. Another recently discovered example of the boron‐containing antibiotics of the tartrolon class is tartrolon E (**4**), produced by the symbiotic cellulose‐degrading bacteria, *Teredinibacter turnerae*, which can be found in the gills of marine shipworms. Tartrolon E (**4**) shows antibacterial activity against *Pseudomonas aeruginosa* and methicillin‐sensitive and methicillin‐resistant *S. aureus* (MRSA) [18]. The mechanism underlying antimicrobial activities of tartrolon E still needs to be investigated further, although one may assume that it also acts as an ionophore [19]. It is worth noting that resistance already exists against such naturally occurring antibiotics. For example, Rolf Müller and colleagues were able to show that *Listeria monocytogenes* possesses an ATP‐binding cassette‐type multidrug resistance transporter, named TimAB which contributes to the resistance against tartrolon antibiotics [17].

**FIGURE 2:**
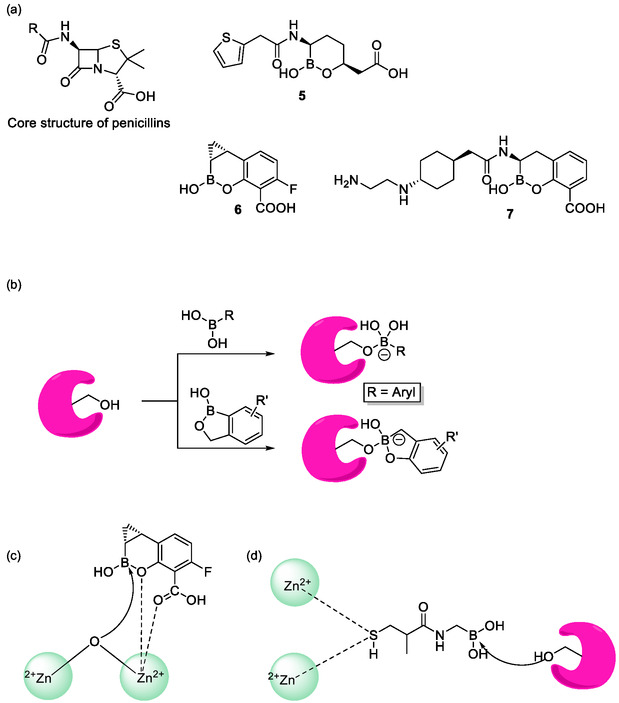
Chemical structures of *β*‐lactamase inhibitors and different mechanisms of *β*‐lactamase inhibition. (a) Structures of vaborbactam (**5**), QPX7728 (**6**), taniborbactam (**7**) and core structure of penicillins. (b) Inhibitors binding at the active site. (c) Inhibitors coordinating zinc. (d) Inhibitors employing both modes of action.

Notably, these natural boron‐containing antibiotics are based on esters of boronic acid, and the ensuing tetravalent boron complexes with oxygen‐based ligands may therefore be considered as representatives of the inorganic side of this semi‐metal. Employing organic synthesis, more eloquent compounds with classical covalent boron‐carbon bonds and covalent boronic acid esters are possible and have been explored extensively. The antimicrobial compounds shown in Figure 2 represent such aromatic derivatives of boronic acid with a covalent boron–carbon bond. Some of them possess a free boronic acid moiety, while others are (cyclic) esters of boronic acid. There are, for instance, several boron‐containing compounds specifically designed to tackle antibiotic resistance. One example is provided by the boronic acid‐based class C *β*‐lactamase inhibitors, which was designed by Waley and his coworkers at Oxford in 1983 [20]. Boronic acid‐based *β*‐lactamase inhibitors mimic the *β*‐lactam structure and can inhibit *β*‐lactamases by covalently binding to the active site serine and/or coordinating the zinc of zinc‐dependent metallo‐*β*‐lactamases (Figure 2). This chemistry, in essence, represents a ligand exchange reaction at the boron atom whereby the HO^−^ ligand is replaced by a serine (Figure 2b). Thus, unlike in the natural ionophores based on boronic esters, the boron atom in these aromatic boronic acid derivatives is not just structurally yet actively involved in the inhibitory action. The combination of suitable *β*‐lactamase inhibitors with *β*‐lactam antibiotics is one of the most successful methods to address *β*‐lactamase‐mediated drug resistance [21]. To this day, vaborbactam **5** is the only FDA‐approved boron‐containing *β*‐lactamase inhibitor which has been developed in 2015 by Hecker et al*.* at *Rempex Pharmaceuticals Inc.* [22, 23]. While most *β*‐lactamase inhibitors only inhibit small sets of *β*‐lactamases, vaborbactam has been developed to serve as a broad‐spectrum inhibitor with specific potency against *Klebsiella pneumoniae* carbapenemase, a class of enzymes capable of inactivating nearly all *β*‐lactams [24]. Notably, a combination of meropenem and vaborbactam has been approved by the FDA in 2017 for the treatment of patients with complicated urinary tract infections [23].

Since vaborbactam lacks the ability to inhibit metallo‐*β*‐lactamases, Hecker et al. reported the synthesis of QPX7728 (**6**), this time at *QPex Biopharma Inc.* in 2020, which inhibits a broad spectrum of *β*‐lactamases, including metallo‐*β*‐lactamases [25, 26]. Paired with approved *β*‐lactam antibiotics, QPX7728 is currently in Phase I trials for the combinational treatment of serious infections induced by Gram‐negative pathogens [27]. At around the same time, Taniborbactam (**7**) has been developed by VenatoRx Pharmaceuticals bearing a similar scaffold as QPX7728. Taniborbactam (**7**) is the first pan‐spectrum *β*‐lactamase inhibitor which entered clinical trials and has successfully completed the Phase III clinical trial in combination with cefepime for the treatment of complicated urinary tract infections [26]. Intriguingly, the cefepime–taniborbactam combination has shown better antimicrobial activity compared to meropenem in the treatment of complicated urinary tract infections with acute pyelonephritis while exhibiting a similar safety profile as that of meropenem [28].

**SCHEME 1:**
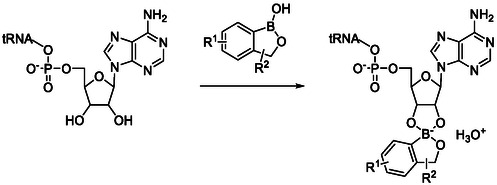
Mechanism of benzoxaborole tRNA‐binding.

Benzoxaboroles form another subclass of aromatic boronic acid derivatives, which consists of compounds featuring a six‐membered aromatic ring fused to a five‐membered oxaborole ring. Members of Benzoxaboroles class generally exhibit a variety of biologically relevant activities including anti‐fungal, anti‐inflammatory, anti‐malarial, anti‐trypanosomal, ectoparasiticide, anticancer, and antibiotic activities, and there are even commercial ‘tool boxes’ available for benzoxaborole‐based drug design [29]. These molecules exhibit prominent antimicrobial activities against Gram‐negative bacteria by inhibiting the editing site of the Leucyl‐tRNA synthetase (LeuRS) enzyme which belongs to the aminoacyl‐tRNA synthetases (aaRSs). In these pathogens, aaRSs provide raw materials for protein synthesis at the ribosome by catalyzing the covalent binding of amino acids to their corresponding tRNA. In essence, aaRSs are crucial for the survival of the pathogen and present a low rate of drug resistance. Some aaRSs are also structurally distinct from their eukaryotic equivalents, making them excellent drug targets. Mechanistically, benzoxaboroles form an adduct with the ribose 2′ and 3′–OH groups of the terminal tRNA^Leu^ adenosine, thereby trapping the tRNA in the editing site of LeuRS (Scheme 1) [21, 30].

**FIGURE 3:**
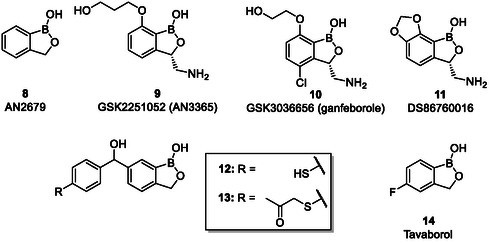
Structures of benzoxaboroles **8**–**14**.

Palencia et al*.* developed a series of 20 benzoxaboroles based on the lead structure AN2679 (**8**; Figure 3) as LeuRS inhibitors [31]. The compounds presented decent biochemical potency as well as excellent whole‐cell activity against *Mycobacerium tuberculosis*. The compounds also exhibited good oral bioavailability which led to the in vivo efficacy not only in acute but also in chronic mouse models of TB. Interestingly, the potency of these benzoxaboroles was comparable to isoniazid, the drug of choice. Epetraborole (**9**; GSK2251052) is another example of benzoxaboroles which inhibits LeuRS function (IC_50_ = 0.31 μmol/l) by binding to the 3′‐terminus of tRNA^leu^ located in the editing active site of LeuRS to form a tetrahedral complex and ultimately leading to bactericidal activity [30, 32]. Epetraborole could not find its way in clinical application due to emergence of microbial resistance in Phase II clinical trial for the treatment of complicated urinary tract infections and inner‐abdominal infections [33]. Currently, ganfeborole (**10**; GSK3036656) has reached in Phase II clinical trials for the treatment of tuberculosis [33]. DS86760016 (**11**) is another promising LeuRS inhibitor which exhibits good activity against extended‐spectrum drug‐resistant *P. aeruginosa* and other multidrug‐resistant Gram‐negative bacteria (e.g., *E. coli*, *K. pneumoniae*) and found its way in preclinical trials [34] Zhou and coworkers have also screened benzoxaborole analogues for their activity against LeuRS in *S. pneumoniae* and identified compounds **12** and **13** bearing thiol moiety with promising activity [35]. Although no antibacterial benzoxaborole has reached the milestone of getting the status of an approved drug yet, the anti‐fungal benzoxaborole tavaborole (**14**) is approved for the treatment of onychomycosis, where it also acts as a LeuRS inhibitor [21].

**FIGURE 4:**
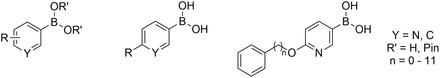
General structures of the aromatic boronic acids with potential antimicrobial activity against MRSA.

Another approach to tackle drug resistance involves the inhibition of the NorA efflux pump, a factor responsible for antibiotic resistance in MRSA. Here again, we find boronic acid derivatives playing a crucial role (Figure 4). These compounds are able to restore the activity of ciprofloxacin and inhibit the resistant *S. aureus* 1199B strain with a minimum modulatory concentration between 0.5 and 128 µg/ml. Boron is crucial for anti‐resistance activity, with pyridine‐3‐boronic compounds showing the best activity [36, 37]. The exact mechanism of inhibition has not yet been unveiled, but an interaction with a serine or threonine residue, as in the case of *β*‐lactamase inhibitors, is quite likely.

A recent strategy to combat bacterial infections involves the utilization of boron clusters, such as carboranes or metallocarboranes, which inhibit the growth of various microbes such as *S. aureus* and *M. tuberculosis* [38, 39]. Boron clusters exhibit diverse morphologies, and consequently, a variety of mechanisms which contribute to an overall excellent antimicrobial activity. Boron clusters, for instance, can disrupt the cell membrane due to their hydrophobic surface. Some other boron clusters, on the other hand, can pass through the cell membrane without disrupting it and inhibit target enzymes via linked organic residues [40]. Furthermore, boron clusters are biologically inert, making it difficult for microbial defence mechanisms to develop resistance [41]. The diverse chemistry of boron clusters is beyond the scope of this review and has been recently reviewed in detail by Fink et al*.* and Price et al. [40, 41].

Zhang et al. have employed a rather interesting approach by constructing a covalent organic framework (COF) based on benzene diboronic acid, which acts as a photocatalyst to promote the release of ROS. The boron‐based COF serves as multifunctional photocatalyst and effectively kills *E. coli* and *S. aureus* under white light irradiation without exhibiting cytotoxicity [42].

Taken together, this brief yet informative excursion into the bioinorganic and bioorganic chemistry of boron supports our notion that semi‐metals may combine the best of both sides, inorganic ionic and complex chemistry on the one side and covalent organic chemistry on the other. This special role and potential of metalliods as interlocutors between inorganic and organic chemistry is nicely exemplified by the benzoxaboroles and becomes even more apparent once we turn to the next semi‐metal in the periodic table, namely silicon.

## 3 Silicon: Silicon Dreams in the Drug Pipeline

**FIGURE 5:**
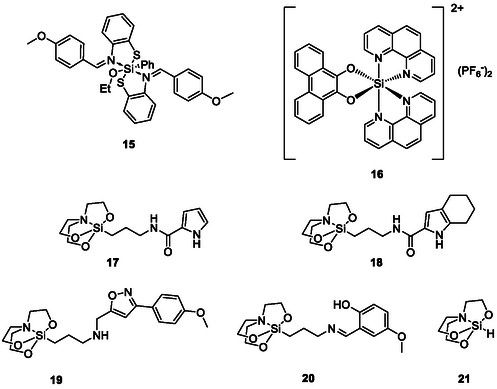
Silicon complexes (**15**–**21**) provide strong antibacterial and antifungal activities.

Indeed, silicon, formally the closest relative of carbon in the periodic table, is an element that stands for a pronounced inorganic chemistry, often associated with silicate SiO_4_^3−^, glass and, as far as physiology is concerned, healthy skin, hair and fingernails [43]. Amazingly, silicon is even the third most abundant trace element in human body, after zinc and iron. Inorganic, silicate‐based molecules are taken up by humans from food, water, and other beverages, such as beer. Since several forms of silicon are rather insoluble, these compounds need to be converted to orthosilicate (OSA) in the acidic environment of the stomach [43, 44, 45]. Once taken up, OSA is incorporated in tissues, such as bones, hair, fingernails, and skin where it is bound to glycosaminoglycans and plays an important role in the formation of cross‐links between collagen and proteoglycans [46]. Although such inorganic SiO_4_^3−^ forms of silicon are quite important for the human body and indeed many other organisms, animals, and plants alike, silicates are not very reactive and thus also hardly of pharmaceutical relevance. Nevertheless, incorporation of silicon into organic drug scaffolds affords several advantages. For instance, the increased bond‐length of Si–C compared to C–C provides the room for different architectures of the scaffolds [47]. Furthermore, the electropositive nature of silicon enhances hydrogen bonding abilities. Last but not least, silicon is able to form octahedral complexes due to its empty d‐orbitals [47]. Complexes of silicon with imine rather than HO^‐^ ligands in some ways therefore form the next, logical step in the development Si‐based therapeutics. Such complexes, for instance compound **15** (Figure 5), have been synthesized and indeed surpass the activity of streptomycin against *S. aureus* [48]. Notably, they also provide antifungal activity comparable to fluconazole. Molecular docking studies suggest that these complexes bind to the active site of DNA gyrase, a type II topoisomerase with the ability to alter DNA topology. Noteworthy, the silicon does not directly interact with the enzyme, and its importance is more of structural nature [48].

In a similar approach, Liao, and coworkers have developed silicon analogues of ruthenium complexes as less toxic and more stable complexes with antifungal activity. The octahedral silicon complex (**16**), for instance, inhibits the growth of *Cryptococcus neoformans* significantly [49].

Still, the famous ‘Silicon Dream’ actually refers less to such inorganic or organometallic complexes of silicon, yet takes advantage of the organic side of this semi‐metallic element. Silicon, chemically often in cahoots with carbon, is not only relevant to plumbers and DIY enthusiasts; although silicon‐based cosmetic and medical implants have gained considerable popularity already, other organic silicon‐carbon compounds have also recently attracted the interest of pharmaceutical chemists.

Various derivatives of silatrane (**17**–**21**), for instance, provide antimicrobial activity against Gram‐negative and, even more so, Gram‐positive bacteria, particularly *Enterococcus durans* and *Bacillus subtilis* [50]. The γ‐aminopropylsilatrane derivative (**20**) shows high antifungal activity agianst *Aspergillus fumigatus*, *Penicillium chrysogenum* and *Fusarium* (MIC = 0.08 μg/ml) while not being antibacterial. Further modifications of the scaffold have already yielded compounds with activity against the unicellular parasites *Giardia lamblia*, *Trichomonas vaginalis*, and *Entamoeba histolytica* [51, 52, 53]. Mechanistically, the silatrane‐moiety can easily be adsorbed to the cell membranes, due to hydrogen bonds and dipole–dipole interactions with polar groups of proteins and lipids, thus finding its way inside the target microbes. Consequently, they primarily serve to transport the organic residue into the cell [54]. In this context, silica nanoparticles should also be mentioned. Although they do not exhibit antimicrobial activity themselves, they can significantly facilitate the transport of drugs across the cell membrane and thus the antimicrobial activity of established drugs [55].

**FIGURE 6:**
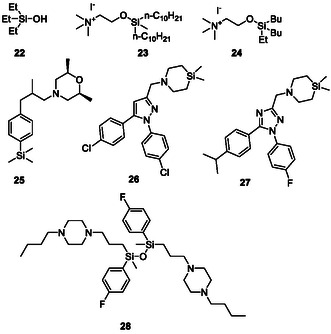
Examples of trialkylsilanoles (**22**) and silicon‐analogues of choline (**23**) and colamine (**24**), fenpropimorph analogue (**25**), rimonabant analogue (**26**), MmpL3 inhibitor (**27**) and the efflux‐pump inhibitor SILA‐421 (**28**).

Whilst silatranes may still be considered as organometallic complexes of Si with O, N and H ligands, organic silicon‐carbon compounds, such as alkylsilanols (Figure 6), clearly represent the organic side of silicon. In 2006, simple trialkylsilanols, such as triethylsilanol (**22**), have been reported to exhibit antimicrobial activity against *E. coli*, *S. aureus*, *P. aeruginosa*, and *Enterococcus faecalis* due to increased lipophilicity and O‐H acidity compared to the corresponding alcohols [56]. Similarly, choline derivatives **23** and **24**, in which the tertiary nitrogen is replaced by silicon, show good activity against Gram‐positive and two Gram‐negative bacterial strains [57]. These compounds also display antifungal activity against *Candida tropicalis* and *Candida albicans*.

Since carbon and silicon share the same group in the periodic table, an isosteric replacement of silicon for carbon seems to be feasible, at least in theory. Indeed, Reddy and coworkers have focused on this interesting strategy in known antimicrobial compounds to yield novel silicon derivatives. Some of the most active resulting silicon compounds are presented in Figure 6 whilst their antimicrobial activities are summarized in Table 1 [58, 59]. Compound **25**, for instance, is an analogue of the antifungal drug fenpropimorph and acts on Δ^14^‐reductase and sterol‐Δ^7^‐Δ^8^‐isomerase, leading to ergosterol depletion and accumulation of the intermediates ignosterol and lichesterol. As a result, the bacterial membrane integrity is compromised which leads to cessation of growth [58]. Regarding the rimonabant analogue **26**, the authors suggest that this compound binds to a mycolic acid transporter called mycobacterial membrane protein large 3 (MmpL3). The increased binding affinity of **26** compared to rimonabant is actually linked to the increased lipophilicity due to the incorporation of silicon [60].

**TABLE 1:** Antimicrobial activities of compounds 25–27.

Compounds	Target microorganisms	MIC (µg/ml)
**25**	*C. albicans* ATCC 24433	0.125
	*C. albicans* ATCC 10231	0.5
	*C. neoformans* ATCC 34664	0.125
	*Candida glabrata* NCYC 388	0.125
	*Candida tropicalis* ATCC 750	0.5
	*Aspergillus niger* ATCC 10578	2
**26**	*M. tuberculosis*	0.031
**27**	*M. tuberculosis* H37Rv	0.03125
	*M. tuberculosis* DR‐TB	0.0625 – 0.5

Based on these studies, Wen et al*.* have developed triazole derivatives as inhibitors of MmpL3 for the treatment of *M. tuberculosis* [61]. In a series of 37 compounds, the most promising drug candidate **27** showed >90% inhibition at concentrations as low as 31.25 ng/ml and with negligible cytotoxicity [61].

In yet another rather elegant approach towards the facet‐rich silicon chemistry, Hegyes et al*.* have exploited the ability of silicates to form Si–O–Si anhydrides and have synthesized the basic disilicate efflux‐pump inhibitor SILA‐421 (**28**; Figure 6) [62]. This compound exhibits excellent bactericidal activity against 21 clinical *M. tuberculosis* isolates in in vitro studies, including drug‐ and multidrug‐resistant strains in a concentration and time dependent manner. SILA‐421 can also enhance the activity of isoniazid (2–4 mg/l SILA‐421 with 0.063 mg/l isoniazid) and rifampicin (2–4 mg/l SILA‐421 with 0.015 mg/l RIF) when applied in combination [63].

To date, there are no studies explaining the fate of silicon‐based antimicrobials after metabolic degradation. However, Tacke et al. were able to demonstrate that sila‐haloperidol, a silicon analogue of the dopamine (D2) receptor antagonist haloperidol, degrades via a completely different pathway than its carbon‐based counterpart, and unlike haloperidol, does not produce any toxic intermediates. It is quite likely that this difference in degradation also applies to the degradation of other silicon analogues [64].

This specific silicate chemistry is certainly worth exploring further, as indeed is most of the biological chemistry associated with this often‐ignored trace element, especially since silicon has, unlike the other metalloids, no intrinsic ‘element‐specific’ toxicity [47].

## 4 Germanium: One of the Forgotten Elements in the Periodic Table

Unlike silicon, germanium is less present in chemistry, daily life, and also biological systems. Indeed, an international conference on germanium chemistry may be held in your garage, and a meeting on biological aspects of this metal may turn out to be a one (wo)man show!

Like the other metalloids discussed so far, germanium can be divided into two main forms: inorganic and organic germanium – and the respective compounds. Whilst inorganic germanium is mostly linked to semiconductor materials and optical fibres, some groups have also reported antimicrobial activities of inorganic germanium materials, such as GeO_2_ incorporated into silicocarnotite ceramics [65]. Interestingly, these ceramics exhibit good antibacterial activity against *E. coli* and *S. aureus* with the GeO_2_ content of 0.05 wt.% or higher [65]. In this context, other forms of GeO_2_ also play a major role in the ‘biochemistry’ of germanium. Inorganic MgO‐GeO_2_ nanocomposite powders, for instance, exhibit antimicrobial activity against *E. coli* and *S. aureus* [66]. Furthermore, GeO_2_ can be incorporated in ternary silicate glass structures, yielding an antimicrobial glass which can be used for dental applications [67].

**FIGURE 7:**
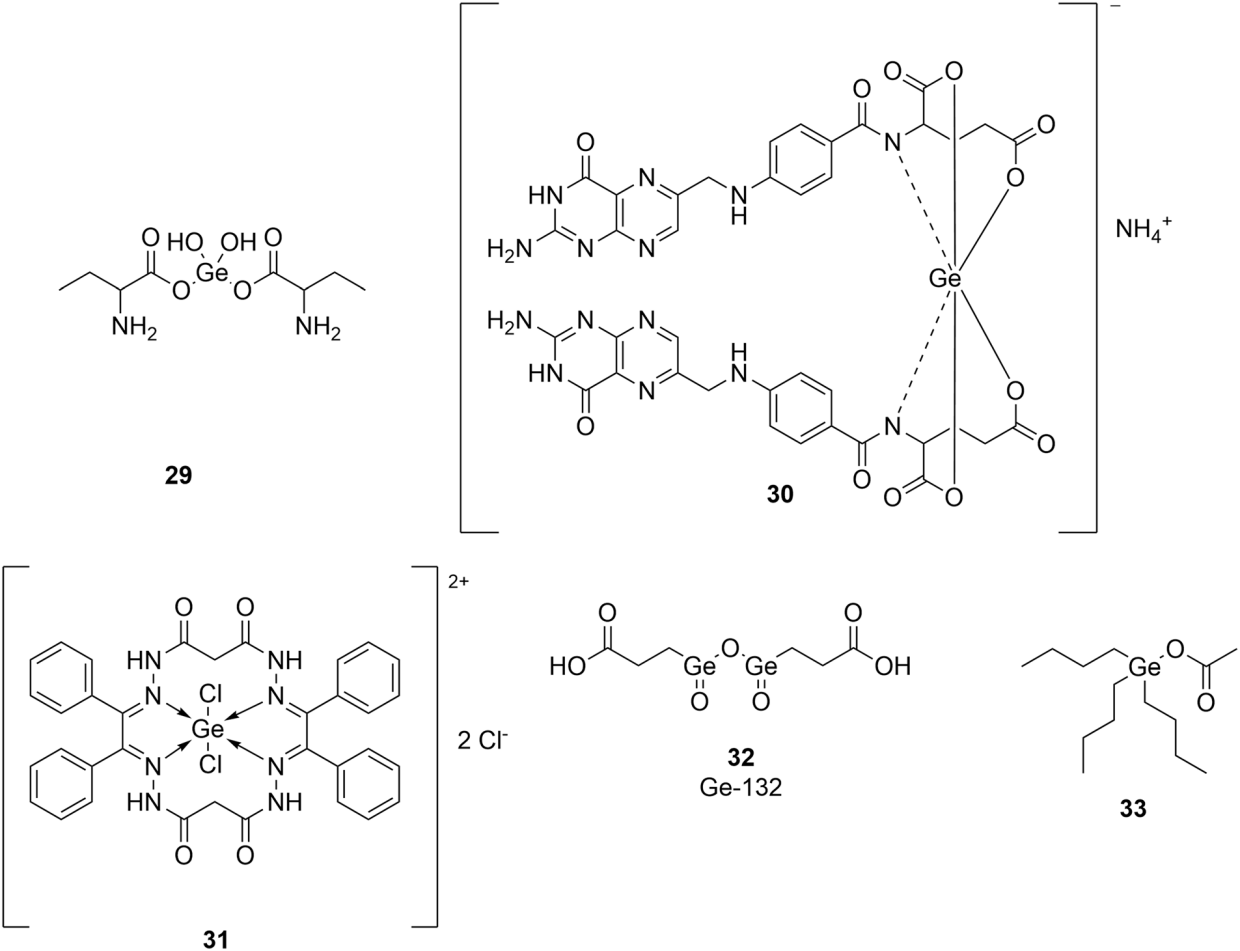
Structures of GeO_2_ in complex with 2‐aminobutanoic acid (**29**), Ge(IV) folic acid complex (**30,** proposed structure) macrocyclic Ge (IV) complex (**31**), Ge‐132 (**32**), and tributylgermanium acetate (**33**).

Moreover, a chitosan‐PVA‐germanium dioxide (Cs‐PVA‐GeO_2_) composite exhibits broad‐spectrum antibacterial activity against Gram‐positive (*S. aureus*, *E. faecalis*) and Gram‐negative (*E. coli*, *P. aeruginosa*) pathogens. As for the underlying mechanism(s) associated with this activity, and based on molecular docking studies, the authors propose strong binding affinities of Cs‐PVA‐GeO_2_ with key bacterial enzymes, including DNA gyrase, penicillin‐binding proteins and dihydropteroate synthase, suggesting a multi‐target inhibitory mechanism [68]. Similarly, GeO_2_ in the form of a typical organometallic complex with 2‐amino‐3‐hydroxybutanoic acid (**29**; Figure 7) provides both bacteriostatic as well as bactericidal activities against *E. coli*, *P. aeruginosa*, *K. pneumoniae*, *S. aureus*, *E. faecalis*, and *Acinetobacter baumannii*, which indeed are interesting targets since they have developed a pronounced antibiotic resistance [69]. Also, a complex of folic acid and Ge(IV) (**30**; Figure 7) shows an increased antibacterial and antifungal activities when compared to the free ligand [70]. It is assumed that these metal ions enhance the antibacterial and antifungal activities by increasing lipophilicity, thus facilitating the penetration of the metal complex across the cell membrane [70]. Furthermore, monometallic complexes of Ge(IV) with macrocyclic ligands, such as complex **31** depicted in Figure 7, show a broad spectrum of antimicrobial activity against both Gram–positive and Gram–negative human pathogenic bacterial isolates [71].

In the field of (mixed) organometallic germanium chemistry, a number of germanium‐containing organotin(IV) compounds with the general formula (Me)_2_Sn(OCOCHR^3^CHR^2^GeR^1^_3_)_2_, where R^1^ = Ph, 4‐MeC_6_H_4_, R^1^ = N(CH_2_CH_2_O)_3_, R^2^ = Ph, H, Me, 4‐MeOC_6_H_4_, 4‐ClC_6_H_4_, 4‐MeC_6_H_4_ and R^3^ = Me, have been screened against various fungal strains such as *Trichophyton longifusus*, *C. albicans*, *Aspergillus flavus*, *Microsporum canis*, *Fusarium solani*, and *Candida glaberata*. It was found that these compounds, in concentrations of 400 μg/ml, are active against these fungi, except for *A. flavus* [72, 73].

With regard to organic germanium compounds, to date, not many compounds with biological activity have been reported. Nonetheless, *bis*‐(carboxyethylgermanium) sesquioxide (Ge‐132; **32**; Figure 7) may be mentioned in this context. This compound was reported first by Mironov et al*.* in 1967, and later Asai and coworkers discovered its potential as a drug [74]. Several in vivo studies have shown that Ge‐132 exhibits antiviral, analgesic, antitumorigenic, and antioxidant activities [75]. Concerning Ge‐containing organic antimicrobials, trialkylgermanium acetates, such as tributylgermanium acetate (**33**), inhibits bacterial and fungal growth of several strains in concentrations of 2–5 µg/ml, although the presence of blood or blood serum in the nutrient medium can counteract this effect [76, 77]. Mechanistically, these compounds seem to follow the similar mechanism of action like their tin‐analogues while being less toxic. These compounds are expected to interfere with the oxidative phosphorylation through the inhibition of F_1_F_O_‐ATPase and disrupt the membrane of bacteria [78, 79, 80]. Furthermore, a soft ligand exchange with cysteine‐containing proteins is conceivable as a mechanism, but there is a lack of scientific evidence. In any case, the specific antimicrobial potential and underlying mechanisms of this class of compounds have not been thoroughly investigated yet.

Taken together, these findings highlight the fact that when it comes to pharmaceutical applications, germanium is a promising, albeit somewhat neglected metalloid element. Still, it may be incorporated into novel antimicrobial drugs, either as an inorganic ion in complexes, as active ingredient of metalloorganic or indeed as main player in organic compounds. In this context, the field of organic germanium compounds has been neglected in the search for new antimicrobials.

## 5 Arsenic: Old Cures and New Challenges

Indeed, moving down the periodic table brings us into ever more ‘metallic’ territory, also for the metalloids, and this is often reflected in the mode(s) of action which become(s) more metal and thus complex‐like. This also holds true for arsenic, one of the most traditional elements in ancient and medieval medicine. Arsenic was described first by Albertus Magnus (1200–1280) around the year 1250 and thus is the only element in the periodic table which has been discovered by a renowned Philosopher. Its initial applications have been less philosophical though, as some of its compounds can be quite poisonous and have also been used as such throughout the centuries. Curiously, arsenic (As) as an element is non‐toxic to humans. Even after the ingestion of 10 g of elemental As, the patients in question survived without any severe complications [81]. Intriguingly, its namesake arsenic (As_2_O_3_) is a notorious toxin, even in very low doses of 1–2.5 mg/kg [82].

The infamous toxicity of As_2_O_3_ (arsenic trioxide) is primarily attributed to its covalent interactions with individual cysteine residues, cysteine clusters, zinc finger motifs, and RING finger domains in proteins. These interactions not only disrupt protein function but also contribute to the development and progression of various diseases [83]. Intriguingly, these same properties also make As_2_O_3_ a promising candidate for antimicrobial applications; for instance, incorporating As_2_O_3_ into polymer composites has shown to effectively inhibit bacterial growth of *E. coli* and *S. aureus* [84].

**FIGURE 8:**
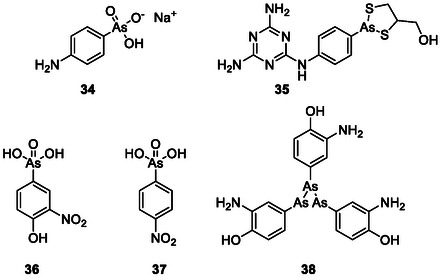
Structures of different historically used arsenic drugs (**34–** **38**). Arsphenamine (**38**) is depicted in its trimeric form as proposed by Lloyd et al. [85].

In the early 1900s, organic arsenicals became a prosperous field in medical research with an astonishing number of approximately 32,000 As compounds being synthesized over the years. Some of these compounds exhibit antimicrobial activities and have also been used as medications. One example is the pentavalent organic arsenical atoxyl (Figure 8; **34**), which was used in the early 1900s by Robert Koch (1843–1910) to treat sleeping sickness yet caused optic atrophy due to its high arsenic content [86]. Nonetheless, the organic arsenic compound melarsoprol (**35**), which was developed in 1949, is still in use today to cure sleeping sickness caused by *Trypanosoma brucei rhodesiense*. It targets trypanothione, which is found exclusively in trypanosomes. Melarsoprol forms an adduct with the thiol groups of two trypanothione molecules. This adduct can no longer cross the cell membrane and thus accumulates in the parasite. Furthermore, melarsoprol acts as an inhibitor of trypanothione reductase. Together, these two mechanisms lead to the loss of the parasite's ability to reduce oxidative stress [87]. In poultry farming, the use of atoxyl and the related synthetic aromatic arsenicals roxarsone (**36**) and nitarsone (**37**) to prevent *Coccidia* and *Histomonas* infections is still common [88]. These compounds disrupt the DNA repair process and energy metabolism by inhibiting ATP synthesis of the parasites [89].

More famous than these organic arsenic compounds is Paul Ehrlich's (1854–1915) “magic bullet”, arsphenamine (**38**), also known as Salvarsan. This unusual tri‐ or pentacyclic structure was used from 1910 onwards to treat human syphilis until it was replaced by penicillin in the 1940s [90, 91]. Arsphenamine itself serves as a prodrug which is oxidized in vivo. Although the mechanism of action is still unclear, it can be assumed that cysteine bonding plays an important role, since As(III) has a high affinity for thiols [85, 92, 93].

Despite the phasing out of most of these early arsenic compounds, the pharmaceutical interest and fascination with this element have never entirely faded. And indeed, even Nature itself provides some unusual arsenic compounds with a pronounced antimicrobial activity.

**FIGURE 9:**
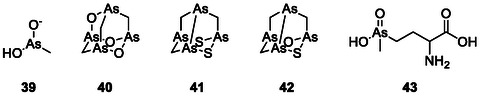
Structures of naturally occurring As‐compounds (**39–** **43**) which provide pronounced antimicrobial activity.

A simple yet amazing natural As‐containing organic product is the highly toxic antibiotic methylarsenite (MAs(III); **39**; Figure 9), which is produced by methylation of inorganic As(III) by the enzyme As(III) *S*‐adenosylmethionine (SAM) methyltransferase, termed ArsM in microbes and AS3MT in animals. The methylation of As is catalyzed by four conserved cysteines [94]. Just like arsinothricin, which will be discussed later, it is produced by soil bacteria to kill other bacteria. Due to the high reactivity of MAs(III), no single target could be identified that applies in every cell [95]. Nevertheless, one target for MAs(III) was recently identified in *Shewanella putrefaciens* 200 [96]. MAs(III), unlike inorganic As(III), effectively inhibits the enzyme MurA (uridine diphosphate (UDP)‐*N*‐acetylglucosamine enolpyruvyl transferase) [95]. MurA is a cytoplasmic enzyme involved in the synthesis of the key precursor of the peptidoglycan, UDP‐*N*‐acetylmuramate (UNAM), which is only essential to prokaryotes [97]. This makes MurA an excellent target for antibiotics. So far, only fosfomycin is approved as an antibiotic that acts against MurA. Fosfomycin inhibits MurA by alkylation of the highly conserved catalytic cysteine residue in the active site. In some microbes, the relevant cysteine residue is replaced by an aspartate, leading to resistance to fosfomycin [98]. MAs(III) could possibly overcome this resistance since it still shows activity against the Cys‐to‐Asp mutants, indicating a different mechanism of inhibition, most likely by binding to a different cysteine residue [96, 99]. Thence MAs(III) could be the starting point of a new generation of antibiotic drugs.

This brings us to one of the most fascinating natural As‐compounds, arsenicin A (2,4,6‐trioxa‐1,3,5,7‐tetrarsatricyclo [3.3.1.13,7] decane; **40**) (C_3_H_6_As_4_O_3_) from the marine sponge *Echinochalina bargibanti*, where As literally holds the key to a complex adamantane system and a pronounced antimicrobial activity, especially against *S. aureus*, *E. coli*, and *C. albicans* [100]. The adamantane bridgeheads in arsenicin A are replaced by As and three methylene groups are replaced by oxygen. Even though research on arsenicin A has been more focused on its anticancer activity, arsenicin A, and the two sulfur‐containing metabolites arsenicin B (**41**) and arsenicin C (**42**) also exhibit strong antimicrobial activities, especially against *S. aureus* [101].

L‐AST but not least, and in addition to these complexes, new impetus has been provided by the discovery of organic As compounds in microorganisms, such as the recently discovered *pentavalent* arsenic (V)‐containing arsinothricin (2‐amino‐4‐(hydroxymethylarsinoyl)butanoate, L‐AST; **43**), produced by the rice rhizosphere microbe *Burkholderia gladioli* and exhibiting broad‐spectrum antibiotic activity while possessing acceptable cytotoxicity. The mechanism underlying the activity of this compound is rather interesting, as it seemingly serves as a non‐proteogenic analogue of glutamate, thereby inhibiting glutamate synthase leading to a potent broad spectrum antimicrobial activity [88]. Notably, L‐AST inhibits the growth of Gram‐negative bacteria more than Gram‐positive bacteria. It also inhibits the growth of *E. coli* more strongly compared to the inorganic As(III) and almost similar to that of highly toxic trivalent methylarsenite (MAs(III)). The high toxicity of L‐AST is particularly interesting, since pentavalent arsenicals are generally much less toxic than their trivalent counterparts, making L‐AST the only known pentavalent arsenic compound, apart from thiolated species, which exhibits such a pronounced toxicity [102]. Intriguingly, L‐AST, compared to its phosphorus analogue L‐PPT, which differs just by one atom (P instead of As), also provides better antimicrobial activity, especially against the pathogenic mycobacterial strain *M. bovis* [88] Moreover, L‐AST effectively inhibits the growth of carbapenem‐resistant *Enterobacter cloacae* (ATCC BAA‐2341), classified within the highest priority category of the World Health Organization (WHO)'s global priority pathogens list. In comparison, other glutamine synthetase inhibitors (L‐PPT and methionine sulfoximine) fail to affect the growth of *E. cloacae*. The efficacy of L‐AST against the resistant microbeshighlights its potential as a promising candidate for the development of novel antimicrobial drugs.

Despite having interesting antimicrobial activities, the As containing compounds face a major challenge due to narrow therapeutic windows (therapeutic indices of 2, 10, and 50–100 for atoxyl, arsphenamine, and melarsoprol, respectively), severe side effects and resistances resulting from widespread uses in animal breeding [103, 104, 105].

## 6 Antimony: Antimicrobial and Traditionally Worth its Money

Unlike the more synthetic metalloid compounds, many of which are currently under pre‐clinical investigation, the use of naturally occurring inorganic antimony compounds as antimicrobial agents dates back as far as the Egyptians and Assyrians, who used elementary antimony and antimony trisulfide as a treatment for fevers and skin irritation [106, 107, 108]. A modern inorganic approach to exploit the antimicrobial properties of antimony involves the utilisation of antimony‐doped tin (IV) oxide nanoparticles (ATO NPs). These ATO NPs are capable of significantly inhibiting the biofilm formation by uropathogenic *E. coli* (UPEC), *S. aureus*, and dual‐species biofilms, as well as reducing their main virulence attributes, such as the cell surface hydrophobicity of UPEC and haemolysis of *S. aureus* [109].

**FIGURE 10:**
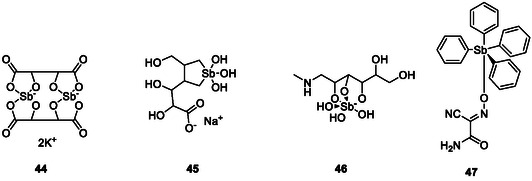
Structures of antimony potassium tartrate (**44**), sodium stibogluconate (**45**), meglumine antimonate (**46**), and tetraphenyl‐antimony(V) cyanoximate (**47**) [110, 111].

Regarding organic antimony compounds, a rather infamous compound is antimony potassium tartrate (Figure 10; **44**), an Sb(III) complex also known as tartar emetic [112]. It was promoted by no one less than Paracelsus (ca. 1494–1541) as an agent against ulcers and was said to have cured King Louis XIV of France (1638–1715) from typhoid fever. Nonetheless, it is also known for its severe side effects, including vomiting, diarrhoea, hepatitis, renal failure, red cell toxicity, cardiovascular collapse, and even death, which brings us to a major drawback of antimony‐containing drugs. Most of them provide severe side effects and toxicity, which dramatically limit their medical uses [112].

Nonetheless, some antimony‐containing drugs are still in use today, especially in the field of neglected tropical diseases, such as leishmaniasis [113]. Antimony potassium tartrate (**44**) has been used until the 1970s for the treatment of schistosomiasis [114, 115, 116]. And indeed, for the treatment of leishmaniasis, the pentavalent antimonials sodium stibogluconate (**45**) and meglumine antimonate (**46**) are still in use today, even though they are not the first choice.

Interestingly, the mechanisms and modes of actions underlying these activities have been studied in more detail, and there are models of activity now for antimony‐based drugs available which so far are notably lacking for many of the other metalloid‐based molecules . The first model for the antimicrobial activity of pentavalent antimonials assumes that their +5 forms are being reduced within the organism to the +3 oxidation state, and that these reduced compounds exhibit a higher toxicity, due to their ability to bind to thiols which in turn act as soft ligands for Sb^3+^ [117]. They may, for instance, inhibit the reduction of trypanothione, an analogue of glutathione solely present in some parasitic protozoa, as antimony might bind to the sulfur atom of the catalytic Cys of trypanothione reductase [117]. Simultaneously, Sb(III) actively decreases the thiol buffering capacity of the parasites by inducing rapid efflux of intracellular trypanothione [118]. As a result, reactive oxygen species (ROS) can not be reduced by the parasite, eventually leading to cell death [117, 119, 120]. Furthermore, pentavalent antimonials may inhibit the expression of genes needed for the survival of the parasite [120]. A second model suggests that type I DNA topoisomerase of the parasite is directly inhibited by stibogluconate and not by meglumine antimonate , indicating that antimony itself is not involved in the mechanism [121]. Pentavalent antimonials have also been reported to bind to ribonucleosides and reduce the levels of ATP and GTP [122]. In short, they cut a major pathway for energy generation and, once again, induce oxidative stress [120].

As for the underlying chemistry is concerned, each of the pentavalent antimonials features antimony in an SbO_6_ environment, yet recent studies have shown that Sb‐C bonds are favourable when compared to such Sb‐O bonds in terms of reducing side effects whilst increasing activity. Not surprisingly, this has led to renewed interest in antimony‐based drugs. Gerasimchuk et al., for instance, have synthesized a series of organic tetraphenyl‐antimony(V) cyanoximates, which exhibit antimicrobial activity [123]. The tetraphenyl‐antimony(V) cyanoximate SbPh_4_ACO (**47**) inhibits the bacterial pathogens including *P. aeruginosa*, *E. coli*, and *S. aureus* with MIC values ranging from 50 to 100 µg/ml, whilst not being cytotoxic*.* The presence of a lipophilic organometallic fragment provides favourable kinetics for the intake of tetraphenyl‐antimony(V) cyanoximates into cells, which facilitates the delivery of biologically active cyanoxime [123]. Although SbPh_4_ACO (**47**) has been shown to disrupt the bacterial membrane yet the underlying mechanism still has to be investigated [106].

If, when and how such antimony drugs and materials may reach clinical trials and practical applications are, of course, relevant questions and one needs to see if antimony‐based drugs can indeed make it again into the clinics or not.

## 7 Tellurium: The Most Metallic of the Lot

This brings us to the – arguably – last and perhaps most down‐to‐earth and metallic of the semi‐metals, as we shall not include Bi and ignore Po and At because of limited relevance and abundance, respectively. Even though the antibiotic properties of Te have been known in the past, this element can be considered as another ‘forgotten’ player in drug development [124]. Interestingly, tellurium is also found in form of tellurocysteine and telluromethionine in proteins of bacteria, yeast, and fungi [125].

Inorganic salts of tellurium can be rather toxic, and historically, TeO_3_^2−^ was used extensively in research – albeit not in medicine – to inhibit the growth of many microorganisms as a “benchmark antibiotic” similar to ciprofloxacin and fluconazol today. Alexander Fleming (1881–1955), for instance, compared the antibacterial activities of penicillin and TeO_3_^2−^ and found that most penicillin‐insensitive bacteria are sensitive to TeO_3_^2−^ and *vice versa*, indicating a certain selectivity of tellurite [124].

Today, inorganic tellurium salts such as TeO_3_^2−^ and TeO_4_^2−^ have mostly vanished from the Life Sciences altogether. TeO_3_^2−^ may not be the most sophisticated drug candidate, still it can enhance the efficacy of antibiotics, possibly in combination therapies whilst reducing antibiotic burden. Therefore, TeO_3_^2−^ may enter the scene again as a player in a promising strategy for combating drug‐resistant bacterial infections, yet this needs to be investigated and studied in the future [126].

**FIGURE 11:**
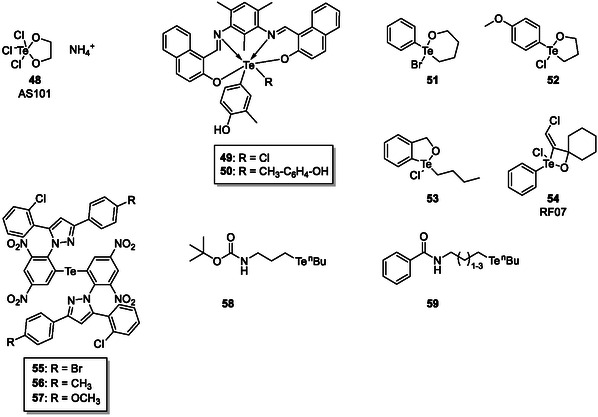
Complexes of tellurium (**48**–**50**) as well as organotellurium compounds (**51**–**59**) provide antimicrobial activity.

As far as the mode(s) of action of TeO_3_^2−^ and its derivatives are concerned, it is likely that they interact with cysteine residues in proteins and enzymes, by complex formation and potentially also by oxidation. Indeed, tellurium, as the most metallic of the metalloids, tends to form complex‐like structures, and the immunomodulator ammonium trichloro[1,2‐ethanediolato‐*O*,*O′*]‐tellurate (AS101; **48**; Figure 11) is one prominent example within the community. AS101 has been found to be active against several parasitic, viral, and bacterial infections, including, for instance, *Leishmania* [127, 128]. In *Enterobacteriaceae* species AS101 damages the cytoplasmic membrane and the pumps connected to it provoking the influx of sodium and potassium ions into the cell and leakage of ATP, nucleotides, and proteins [128]. Even though several tellurium compounds are considered highly toxic, AS101 is in fact non‐toxic, demonstrating that it is possible to develop non‐toxic tellurium‐based drugs [129]. More recently, a set of novel organotellurium(IV) complexes employing a newly developed Schiff base (1,1′‐(((2,4,6‐trimethyl‐1,3‐phenylene)*bis*(azaneylylidene))*bis*(methaneylylidene))*bis* (naphthalen‐2‐ol), HNTD) (**49**,**50**), has been developed and tested for their activities against *Plasmodium* and other microbes. The study has identified different HNTDs to exhibit antimalarial, antifungal, and antibacterial activities [130].

Besides such complexes, organo‐tellurium compounds are also active against parasitic organisms, although their chemical and especially metabolic stability is always in question. *Plasmodium falciparum*, for instance, which causes malaria, is sensitive to naphthoquinone couples with a selenium or tellurium moiety [131]. The rather selective toxicity against plasmodia may be due to the fact many tellurium agents are redox active and they increase levels of oxidative stress, while plasmodia lack certain antioxidant defence systems and hence are especially sensitive to this condition [124]. Tellurium‐based compounds may therefore hold some promise to target organisms with a weak(er) oxidative defence rather efficiently and selectively.

**SCHEME 2:**
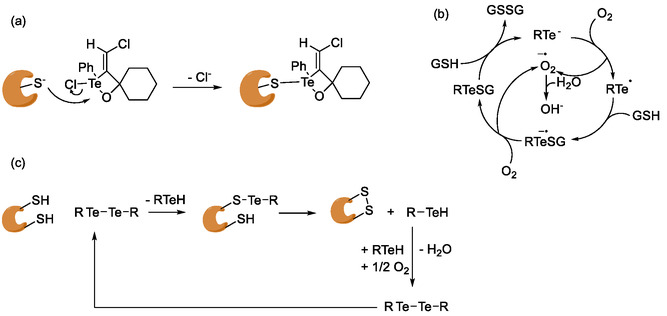
(a) Covalent protein binding of RF‐07 as proposed by Cunha et al. [134]. (b) ROS generation through tellurium‐containing compounds. (c) Dimerization of vicinal cysteine residues through ditellurites.

A recent study by Souza et al*.* reports the anti‐protozoal activity of organotellurium compounds especially **51**–**53**, which have exhibited IC_50_ values in the low micromolar range against *L. amazonensis* promastigotes [132]. Moreover, compound RF‐07 (**54)** has been reported to exhibit the biological activity in sub‐micromolar concentrations against *L. chagasi*, with a selectivity index of 10 [133]. Compound RF‐07 is an inhibitor of cysteine protease cathepsin B, which also exists in leishmania [132]. The overall biological activity is most probably achieved by binding covalently to the cysteine residue of the active site of cathepsin B as depicted in Scheme 2a [134].

As far as the antibacterial activity of organotellurium compounds is concerned, Sabti and coworkers have synthesized a series of compounds (**55**–**57**) based on pyrazole derivatives, some of which show better antimicrobial activity compared to amoxicillin [135]. Reis de Sá and coworkers have also synthesized a series of tellurium‐based organic compounds to target the efflux pumps of *C. albicans* in order to overcome fluconazole resistance. Indeed, compounds **58** and **59** have been able to somewhat restore the fluconazole sensitivity of *S. cerevisiae* strains overexpressing *C. albicans* Cdr1p and Mdr1p, by inhibiting CaCdr1p ATPase activity, while exhibiting no cytotoxicity to human cell lines [136].

Although the mechanisms of the antimicrobial activity of the tellurium containing compounds are still unclear, tellurium, as a chalcogen, is highly redox‐active. Thus, tellurium containing compounds are not limited to soft ligand interactions with cysteine yet are also capable of actively generating ROS by reacting with oxygen and gluthatione (GSH) as depicted in Scheme 2b [125]. Furthermore, ditellurides, for instance, can dimerize vicinal cysteine residues in proteins (Scheme 2c).

Apart from the studies presented here, the research on the antimicrobial activities of small‐molecule organotellurium compounds has been rather scarce. In other approaches, tellurium has been implemented into polymeric materials like chitosan‐fabricated tellurium nanoparticles (CS‐Te NPs) or tellurium‐loaded polymeric fibre meshes [137, 138]. The latter approach has yielded a material capable of effectively inhibiting bacterial growth of *E. coli* and therefore can be used as an antibacterial fibre. CS‐Te NPs exhibit strong antimicrobial activity not only against bacteria, fungi, and yeast but also inhibit Gram‐positive and Gram‐negative biofilm formation. Mechanistically, Te NPs are capable of generating ROS through their unique electronic environment. Once in contact with water or oxygen Te can transfer electrons to molecular oxygen, thus forming superoxide anions (O_2_^·−^) and thereby starting a cascade of ROS formation leading to microbial cell death [139].

Taken together, these results highlight the potential of tellurium‐containing compounds as antimicrobial agents. However only one of the presented compounds, namely AS101, has found its way into clinical trials, as a immunomodulating drug, *i.e.* for a non‐antimicrobial application [140].

## 8 Summary and Outlook

This brief and necessarily incomplete look at the six traditional metalloid elements, a few of their more interesting compounds and respective biological activities has underlined the versatility and potential uses of some of them. Together, these semi‐metals combine benefits from the inorganic, ionic and complex chemistry on the one side and the covalent, organic chemistry on the other, and therefore bestow their respective compounds with considerable flexibility and diversity in reactivity and modes of action. Notably, several of these compounds, from boric acid and silicate to arsenic and antimony have been and still are being used in the therapy of certain diseases, such as parasitic infections. Others have been superseded by modern antibiotics and yet sevral others are being in the pipeline for possible resistance‐busting antimicrobial drugs or to fight some of the more neglected and perhaps exotic diseases where mainstream pharmaceutical research has not yet provided efficient solutions.

In the future, one should therefore keep an eye on these metalloids, as they reflect a distinct chemistry between the metals and non‐metals and thus may be able to carry out tasks neither classic metals nor non‐metals can do easily on their own, just like fixing your bathtub with silicone.

## Author Contributions

**Kevin Böhm**: conceptualization (supporting), data curation (equal), validation (equal), writing – original draft (equal). **Muhammad** **Jawad Nasim**: formal analysis (lead), supervision (lead), validation (lead), writing – original draft (supporting), writing – review & editing (supporting). **Claus Jacob**: conceptualization (lead), formal analysis (equal), supervision (lead), visualization (equal), writing – original draft (lead), writing – review & editing (equal).

## Conflicts of Interest

The authors declare no conflicts of interest.

## Data Availability

Data sharing is not applicable to this article as no new data were created or analyzed in this study.
